# Accurate Serodetection of Asymptomatic Leishmania donovani Infection by Use of Defined Antigens

**DOI:** 10.1128/JCM.02620-15

**Published:** 2016-03-25

**Authors:** Aarthy C. Vallur, Caroline Reinhart, Raodoh Mohamath, Yasuyuki Goto, Prakash Ghosh, Dinesh Mondal, Malcolm S. Duthie, Steven G. Reed

**Affiliations:** a Infectious Disease Research Institute, Seattle, Washington, USA; b International Center for Diarrhoeal Diseases Research, Laboratory Sciences Division, Dhaka, Bangladesh

## Abstract

Infection with Leishmania donovani is typically asymptomatic, but a significant number of individuals may progress to visceral leishmaniasis (VL), a deadly disease that threatens 200 million people in areas where it is endemic. While diagnosis of acute VL has been simplified by the use of cost-effective confirmatory serological tests, similar standardized tools are not widely available for detecting asymptomatic infection, which can be 4 to 20 times more prevalent than active disease. A simple and accurate serological test that is capable of detecting asymptomatic L. donovani infection will be useful for surveillance programs targeting VL control and elimination. To address this unmet need, we evaluated recombinant antigens for their ability to detect serum antibodies in 104 asymptomatic L. donovani-infected individuals (qualified as positive for L. donovani-specific antibodies by direct agglutination test [DAT]) from the Mymensingh district of Bangladesh where VL is hyperendemic. The novel proteins rKR95 and rTR18 possessed the greatest potential and detected 69% of DAT-positive individuals, with rKR95 being more robust in reactivity. Agreement in results for individuals with high DAT responses, who are more likely to progress to VL disease, was 74%. When considered along with rK39, a gold standard antigen that is used to confirm clinical diagnosis of VL but that is now becoming widely used for surveillance, rKR95 and rTR18 conferred a sensitivity of 84% based on a theoretical combined estimate. Our data indicate that incorporating rKR95 and rTR18 with rK39 in serological tests amenable to rapid or high-throughput screening may enable simple and accurate detection of asymptomatic infection. Such tests will be important tools to measure L. donovani infection rates, a primary goal in surveillance and a critical measurement with which to assess elimination programs.

## INTRODUCTION

Visceral leishmaniasis (VL or kala-azar) is one of the deadliest and most neglected tropical diseases in the world. It affects the poorest among mostly rural populations where the disease is endemic, with around 300,000 new cases every year (1, 2). Of these, about 90% occur in the Indian subcontinent, Brazil, and East Africa, while VL is an emerging threat in the Mediterranean basin (1).

In the Indian subcontinent, VL is caused by infection with Leishmania donovani. Importantly, VL in the Indian subcontinent has some unique features that appear to make it a candidate for elimination (3, 4). The presence of a single vector, anthroponotic transmission, and a defined, rural area of endemicity have empowered the governments of India, Nepal, and Bangladesh to propose the kala-azar elimination program, which aimed to reduce the incidence of VL to <1 case per 10,000 individuals by 2015 (5). Early case detection, treatment, and integrated vector control are three of the six pillars of the program (5–7). Of these, the practical difficulties plaguing early case detection have been a roadblock in advancing toward the elimination program objectives (8, 9). Recognizing the potential of rK39-based rapid diagnostic tests (RDT), the elimination program has made these tests readily available to clinics and health care workers in regions where VL is endemic, thereby enhancing detection of acute VL cases. A major challenge for the elimination campaign is the large population of asymptomatic L. donovani-infected individuals residing in regions where it is endemic. Such individuals harbor low levels of infection but display no symptoms of VL. Although clearance of the parasites appears to be the typical outcome, these individuals can potentially transmit parasites to others and are at an elevated risk of developing VL (10, 11). Early case detection hinges on identifying asymptomatic individuals and monitoring them periodically. Thus, surveillance, detection, and monitoring of asymptomatic L. donovani infection are absolutely necessary to realize kala-azar elimination.

Existing diagnostic tools are not entirely suitable for detection of asymptomatic infection. Diagnostic methods such as microscopy of splenic aspirates are unethical in asymptomatic individuals and are unsuitable for the surveillance of a large population. The rK39 RDT recommended for confirming VL disease in the Indian subcontinent is not fully reliable for the screening of asymptomatic L. donovani infection (12). At present, an enzyme-linked immunosorbent assay (ELISA) against rK39 and the direct agglutination test (DAT) are commonly used in large surveillance studies for asymptomatic infection in the Indian subcontinent (13–16). Although DAT is effective for detecting L. donovani infection because it offers a broader antigen panel, the use of freeze-dried promastigotes as the detecting antigen can render it susceptible to lot-to-lot variations (13, 17). In addition, DAT is labor-intensive, markedly lower in throughput than a standard ELISA, and most importantly, because it is a visual test, it is extremely difficult to set uniform standards for widespread use.

Detection of asymptomatic L. donovani infection is an urgent need within VL control programs. Given that asymptomatic L. donovani infections are more common than VL, there is a need for simple and standardized tools to provide sensitive, specific, and quantitative results while also facilitating high-throughput screening within regions where the disease is endemic. In an attempt to develop a recombinant antigen-based serological test with these properties for use in the surveillance of asymptomatic infection, we evaluated several Leishmania antigens in an ELISA on serum from likely asymptomatic L. donovani-infected individuals in Bangladesh. Through this process, we selected two recombinant antigens that complemented rK39 ELISA and that were comparable to DAT in detecting asymptomatic L. donovani infection. We discuss our results in terms of a dedicated serological tool to screen areas of endemicity for asymptomatic L. donovani infections in a conventional ELISA or a rapid test format.

## MATERIALS AND METHODS

### Samples.

All samples were collected following approval from the respective ethics committees and after obtaining individual consent forms.

Blood was obtained and serum samples/DNA were prepared from individuals with no history of VL or post kala-azar dermal leishmaniasis (PKDL) residing in the region in Harirampur Union, Trishal subdistrict, Mymensingh district, Bangladesh where VL is hyperendemic as described before (10). Initial consent was obtained from the head of household to screen household members, and then individual written consent was obtained from participants prior to study enrollment. Serum samples from clinically confirmed VL patients were included as positive controls. Serum samples from 46 healthy individuals in the United States who had no history of travel outside of the United States (purchased from Equitech, TX) were used as nonendemic controls (NECs) to establish cutoffs for sensitivity. In addition to the NECs, serum samples from healthy endemic controls (EC) from the Mymensingh district were used. To measure cross-reactivity with other diseases (OD), serum samples from patients with non-VL febrile illnesses from a region where VL is nonendemic (the Philippines) were used. These individuals were defined with no history of VL due to negative responses in DAT and rK39 RDT.

### Initial sample characterization.

Initial serum characterization was conducted using the direct agglutination test (DAT) (KIT Biomedical, Amsterdam, Netherlands) performed according to the manufacturer's instructions at the International Centre for Diarrhoeal Disease Research, Bangladesh (icddr,b) (Dhaka, Bangladesh). Based on a DAT titer of >1,600 in these evaluations, 104 serum samples were designated DAT positive and are referred to as asymptomatic L. donovani-infected individuals in this study. The 104 individuals were also tested with finger prick blood for positivity with the rK39-RDT (KalaAzar Detect; InBios International, Seattle, WA) by investigators from icddr,b, Dhaka, Bangladesh. Healthy endemic controls were defined as DAT- and rK39-RDT-negative individuals from the same area as the asymptomatic individuals. Aliquots of the serum samples were shipped to the Infectious Disease Research Institute (IDRI) and archived.

### Recombinant proteins.

Several tandem repeat (TR) proteins previously identified in a bioinformatics screen and validated as being reactive with VL patient serum samples were evaluated (18). Kinesin-like protein was identified by a liquid chromatography-tandem mass spectrometry (LC-MS/MS) analysis of serum and urine samples from VL patients that was performed using standard procedures at ITSI-Biosciences (Johnstown, PA). Peptide hits were then screened for Leishmania specificity against L. donovani and L. infantum databases using Proteome Discoverer 1.2 and the SEQUEST algorithm. Peptides specific to VL patient samples but not to healthy U.S. samples were considered Leishmania-specific peptides. L. donovani kinesin-related protein, henceforth referred to as rKR95 (GI112293604), was identified by 5 peptide hits in 4 VL serum samples and by 1 peptide hit in 1 VL urine sample and was chosen for further validation after confirming a lack of homology to human counterparts (Table 1). The construct encoding the 742 amino acid sequence that contains all six peptides was synthesized and cloned into pET17a with an N-terminal 6×His tag (Gene Dynamics, Tigard, OR). The sequence verified expression construct was expressed from E. coli strain BL21(DE3) pLys (Invitrogen) and was purified as described previously (18). The purity and integrity of each protein were confirmed by SDS-PAGE. rK39 antigen was made as described previously (19).

**TABLE 1:** Performance of ELISA comprising rTR18, rKR95 and rK39 versus DAT

Antigen(s)	Total no, % (*n* = 104)	No. with DAT titer of <6,400, % (*n* = 23)	No. with DAT titer of >6,400, % (*n* = 81)	% Specificity (*n* = 48)^*a*^
rTR18	34 (33)	4 (17)	30 (37)	94
rKR95	66 (63)	10 (43)	56 (69)	89.5
rTR18 and rKR95^*b*^	72 (69)	12 (57)	60 (74)	83
rK39	82 (79)	14 (61)	68 (84)	87.5
rTR18, rKR95 or rK39^*b*^	87 (84)	16 (70)	71 (88)	83

a Specificity was calculated against DAT-negative endemic controls.

b Theoretical estimate based on the combined sensitivity of the antigens.

L. donovani soluble lysate antigen (SLA) was made by lysing 1 × 10^7^ cultured L. donovani MHOM/80/IN/DD8 promastigotes in the presence of 1× protease inhibitors (Halt protease inhibitor cocktail; ThermoFisher) and 5 mM EDTA by repeated freeze-thawing followed by sonication and by separating insoluble material by centrifuging for 45 min at 15,000 rpm.

### Antibody reactivity by ELISA.

All ELISAs were done at IDRI. High binding 384-well plates (Corning, NY) were coated with 1 μg/ml recombinant protein or 2.5 μg/ml L. donovani SLA in the ELISA coating buffer (eBiosciences, Inc., San Diego, CA) at room temperature for 4 h and were washed with phosphate-buffered saline (PBS)-Tween (PBS-T) before being blocked overnight at 4°C with PBS-T with 1% bovine serum albumin (BSA). Plates were washed as before. Serum diluted 1:200 in PBS-T with 0.1% BSA was then added to each well, and plates were incubated at room temperature for 2 h on a shaking platform. After washing as before, horseradish peroxidase-conjugated anti-human IgG (heavy and light chains) (Invitrogen) diluted 1:8,000 in PBS-T with 0.1% BSA was added to each well and was incubated for 1 h at room temperature. Plates were washed again and developed with SureBlue TMB microwell peroxidase substrate (KPL, Inc., Gaithersburg, MD). To stop the reaction, 1 N H_2_SO_4_ was then added to each well. The optical densities of each well were immediately read at 450 nm (OD_450_) and 570 nm (OD_570_). The final, background-adjusted signal for each sample was calculated by subtracting the 570-nm reading from the 450-nm reading. The cutoff for seropositivity on each plate was defined as the mean signal of normal serum plus 2 standard deviations. Signal to noise ratios were calculated as ELISA signal over cutoff for any antigen. Ratios of >1 were considered positive.

### Sequence alignments.

The BLASTn suite (NCBI) was utilized using the cloned sequences as queries against Leishmania and non-Leishmania genomes or cDNA sequences when genomes were not available.

### Statistical analysis.

One-way analysis of variance (ANOVA) was used to determine the statistical significance between nonparametric data sets to assess the performance of antigens, with a *P* value of <0.05 considered significant. All analysis was done using GraphPad Prism 6 software (GraphPad Prism Inc., San Diego, CA).

## RESULTS

### Preliminary evaluation of antigens in a test for asymptomatic infection.

**FIG 1:**
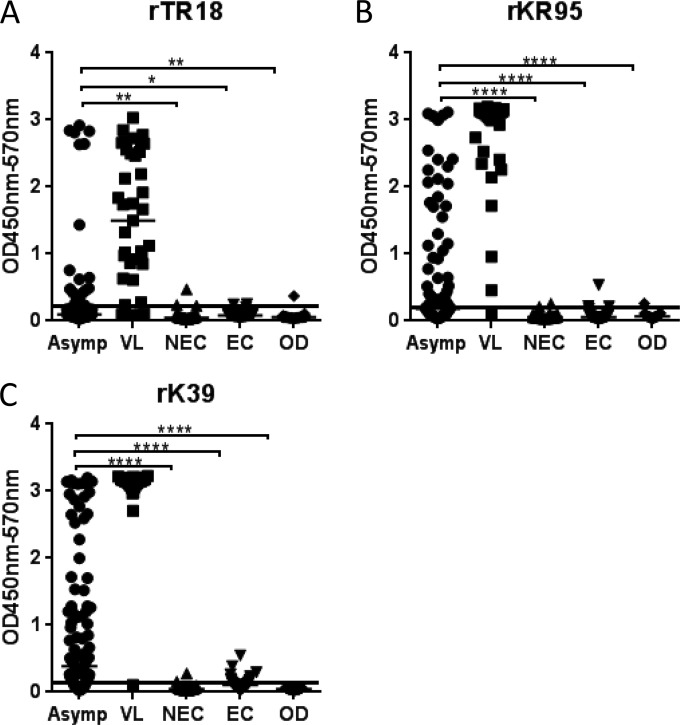
Assessment of the ability of a serological test comprising recombinant proteins to detect antibodies in the serum of asymptomatic infected individuals. Asymptomatic infected individuals were defined as DAT positive, and serum antibodies against the indicated antigens were measured by ELISA. The optical density of each serum sample from asymptomatic individuals (Asymp) (circles), VL patients (VL) (squares), healthy nonendemic controls (NEC) (triangles), DAT-negative healthy endemic controls (EC) (inverted triangles), and controls with other diseases (OD) (diamonds) is plotted. Median optical density is indicated for each group (black bars). The black line intersecting each plot identifies the cutoff above which samples were considered positive. ****, *P* value of <0.0001; * and **, *P* value of <0.05 and <0.01, respectively, as measured by one way ANOVA.

A preliminary panel of serum samples from 24 VL patients, 24 asymptomatic L. donovani-infected individuals from Bangladesh, and 40 NECs was evaluated by ELISA against tandem repeat proteins and rKR95 (data not shown). Of the recombinant antigens examined, only rTR18 and rTR95 displayed the suitable outcome of high reactivity against VL serum, low reactivity against NECs, and various responses in asymptomatic infected individuals (data not shown). To further evaluate the ability of rTR18 and rKR95 to bind antibodies on serum from asymptomatic infected individuals, ELISAs were conducted on an expanded panel of serum samples from Bangladesh, consisting of samples from 104 asymptomatic individuals, 39 VL patients, 48 healthy endemic controls, and 37 patients with other diseases. The individuals were assessed as asymptomatic based on testing positive by DAT, which indicated either current or prior L. donovani infection. For responses against rTR18, a low median signal was observed, indicating that asymptomatic infected individuals possess low levels of antibodies relative to those of symptomatic VL cases from Bangladesh. A considerable number of serum samples from asymptomatic individuals did however demonstrate antigen-specific antibody levels comparable to those of VL patients (Fig. 1A, left). The median signal for responses against rKR95 was also low, but a greater proportion of serum samples from asymptomatic individuals demonstrated anti-rKR95 antibody levels similar to VL patients than those of rTR18 (Fig. 1A, right). The high signal to noise ratios and the significant differences between asymptomatic infected individuals and controls (NEC, EC, and OD) indicated the specificity of the two antigens to L. donovani infection (Fig. 1A).

In contrast, other antigens that were screened for activity against asymptomatic infected individuals displayed high backgrounds, low signals, or both, making them unsuitable for discerning infection (data not shown). Taken together, these data indicate the utility of rTR18 and rKR95 in detecting asymptomatic infection.

### Serological test based on rTR18 and rKR95 is comparable to DAT.

DAT is widely used for surveillance purposes in the Indian subcontinent. Owing to the broad antigen panel of DAT, it is also considered to be a more reliable predictor of asymptomatic infection than the rK39-based tests (13). We considered DAT positivity as the enrolling criteria for the 104 asymptomatic infected individuals from Bangladesh in this study. Positivity by rTR18 was 34/104 (33%) and by rKR95 was 66/104 (63%) (Table 1). Although it had lower sensitivity than rKR95, rTR18 detected 6 asymptomatic serum samples that were rKR95 negative (Table 1).

DAT titers of ≥6,400 were recently shown to indicate a greater risk of progression to VL disease in asymptomatic individuals from the Indian subcontinent (13). Performance of rTR18 and rKR95 was therefore assessed on the asymptomatic individuals further classified as low DAT positives (DAT titer of <6,400) and as high DAT positives (DAT titers of ≥6,400). Positivity results by rTR18 and rKR95 on serum samples that had low DAT results were 4/23 (17%) and 10/23 (43%), respectively (Table 1). Better agreement was seen with serum samples that had high DAT results, with rTR18 detecting 30/81 (37%) and rKR95 detecting 56/81 (69%) (Table 1). Although 26 of the rTR18-positive samples in this group were also positive by rKR95, 4 were positive only by rTR18. The theoretical combination of either antigen detected 60/81 (74%) of the high DAT-positive samples (Table 1). Based on the EC samples, specificity was 94% for rTR18 and 89.5% for rKR95 (Table 1). The signals of the control samples that were positive were close to the cutoff, which indicated very low reactivity.

We were surprised that 21 of the high DAT-positive samples displayed low reactivity to either antigen. Since DAT has a broader panel of antigens derived from whole promastigotes, it is possible that diverse antibodies are contributing to higher positivity by DAT than by the recombinant antigen. To have a comparative panel of antigens to DAT, we used L. donovani SLA, derived from lysed promastigotes in ELISAs on the same serum samples. We have previously reported that lysate is more sensitive than single antigens in detecting asymptomatic infections (10). Surprisingly, 20% of the DAT-positive samples were negative by L. donovani SLA, including 8 high DAT-positive samples that were also negative of rTR18 and rKR95 (data not shown). It is possible that antibody deterioration upon storage or similar factors affected the performance of the ELISAs, which used archived samples compared to DAT, which was done on fresh samples. Hence, it is possible that the true agreement between rTR18 and rKR95 with DAT may be higher than that observed in this study.

### Complementation of rK39 by rTR18 and rKR95.

**FIG 2:**
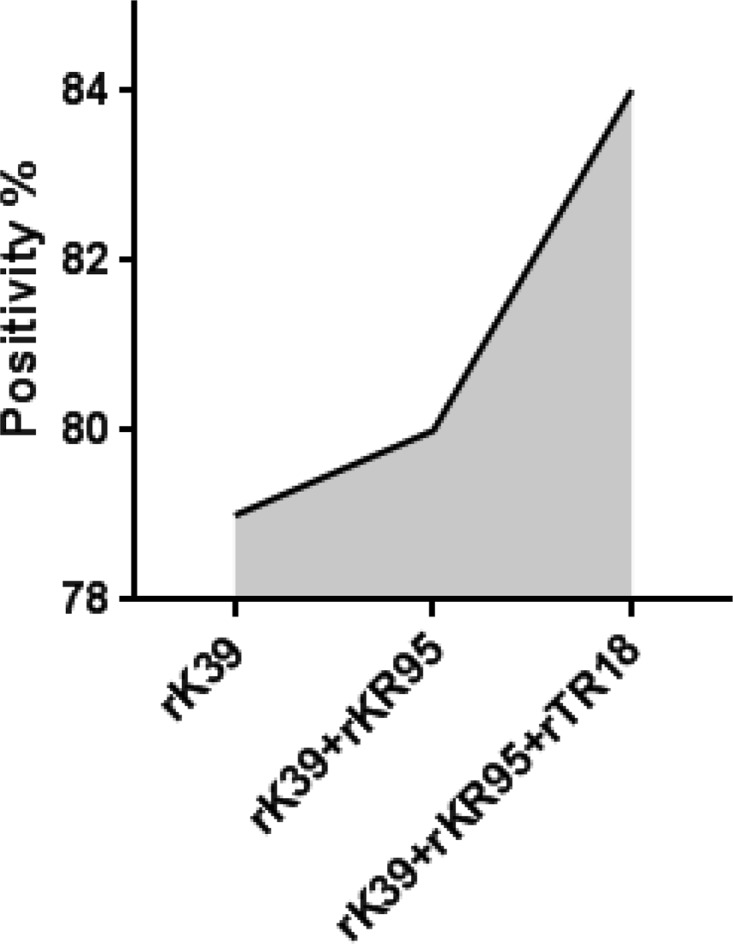
Illustration of incremental augmentation of sensitivity in ELISAs when rK39, rTR18, and rKR95 were considered together.

Tests based on the rK39 antigen are now used in conjunction with clinical exams to confirm VL disease in the Indian subcontinent (20). It has been documented that higher antibody titers against the rK39 antigen are generated in VL patients from the Indian subcontinent than in other regions where VL is endemic (20). Based on this observation, the rK39 ELISA has been used as a quantitative measure of anti-Leishmania antibodies in recent surveillance studies along with DAT (13). We therefore evaluated how positivity against rK39 compared with that against rTR18 and rKR95. A positivity of 82/104 (79%) by rK39 ELISA was observed on the study samples (Table 1). Of the rK39 ELISA-negative samples, rTR18 and rKR95 detected 5/22 (Fig. 1C and Table 1). When data for all three antigens were considered together, an incremental increase was suggested that would provide a combined positivity of 87/104 (84%) for the detection of asymptomatic infected samples, which is higher than that achieved by either rK39 alone (79%) or by the theoretical combination of rK39 and rKR95 (69%) (Fig. 2). rK39 ELISA trends were similar to those of the two antigens when sorted based on DAT titers, with 14/23 (61%) positivity among low DAT titer samples and 68/81 (84%) positivity among high DAT titer samples (Table 1). When positivity by rK39, rKR95, and rTR18 was considered together, 70% of low DAT samples and 88% of high DAT samples were detected, which is more than that achieved by rK39 alone (Table 1). Our data indicate that a test comprising rTR18 and rKR95 in combination with rK39 will be more effective in detecting asymptomatic infection.

### rTR18 and rKR95 are conserved and Leishmania specific.

Highly immunogenic TR proteins have been reported to be conserved in sequence (21). Indeed, genetic alignment indicates that rTR18 is highly conserved across Leishmania species, not just in those that cause VL (Table 2). The other selected antigen, rKR95, is also highly conserved across Leishmania species causing VL and cutaneous leishmaniasis (CL) (Table 2). The high level of conservation of each protein across Leishmania species suggests that they serve important functions at some life stage of the parasite. We also assessed sequence similarity with other pathogens that are prevalent in regions where leishmaniasis is endemic, that share sequence homology with Leishmania, or that cause febrile illnesses that can be confused with VL. No identity was seen in the genera Mycobacteria or Salmonella that is prevalent in regions where VL is endemic. No similarity was seen with Trypanosoma species that cause African trypanosomiasis, and a segment representing approximately half (47%) of the mRNA sequence of rKR95 had 79% sequence identity with a kinesin-like protein in Trypanosoma cruzi, which causes Chagas disease in South America (Table 2). However, when serum samples from patients with Chagas disease were evaluated by rKR95 ELISA, no reactivity was observed (data not shown). Sequence similarities were not reported against any of the evaluated organisms for rTR18. Thus, we conclude that rTR18 and rKR95 are conserved in Leishmania species, and antigen-specific antibody responses are limited to Leishmania infection.

**TABLE 2:** Identities of selected antigens^*a*^

Antigen	Sequence conservation		Mol wt (kD)	Function
	Organisms	Identity (%)		
rKR95	L. donovani	100	95	Conserved motor protein
	Leishmania infantum	100		
	Leishmania major	99		
	Leishmania mexicana	99		
	Leishmania braziliensis	93		
	Leishmania tropica	NA^*b*^		
	Leishmania aethiopica	NA		
	Non-leishmania			
	Plasmodium	None		
	Trypanosoma cruzi	79^*c*^		
	African trypanosomes	None		
	Salmonella	None		
	Mycobacteria	None		
rTR18	L. donovani	100	18.6	Hypothetical protein
	L. infantum	100		
	L. major	94		
	L. mexicana	89		
	L. braziliensis	NA		
	L. tropica	NA		
	L. aethiopica	NA		
	Non-leishmania			
	Plasmodium	None		
	Trypanosoma cruzi	None		
	African trypanosomes	None		
	Salmonella	None		
	Mycobacteria	None		

a Gene sequences for rTR18 and rKR95 were aligned to the published genomes of the indicated Leishmania species and other organisms using BLASTn. Molecular weights and hypothesized functions of rTR18 and rKR95 are noted. Alignments were done against the taxid for Mycobacteria (taxid: 85,007 including Mycobacteria tuberculosis, Mycobacteria leprae, Mycobacteria ulcerans, Mycobacteria avium, and Mycobacteria smegmatis), Salmonella (taxid: 590 including Salmonella enterica, Salmonella typhi, and Salmonella paratyphi), and Plasmodium (taxid: 5,820 including Plasmodium vivax, Plasmodium malariae, Plasmodium falciparum, and Plasmodium ovale).

b NA, not applicable.

c Alignment observed only between 47% of the putative cDNA sequences.

## DISCUSSION

VL is caused by L. donovani infection in the Indian subcontinent, and efforts to eliminate the disease center on reducing transmission and progression to acute disease. A major difficulty in defining prevalence and incidence of infection is the lack of an easy to use and, above all, accurate tool for the detection of infected individuals. To develop such a test, a gold standard against which a candidate test can be evaluated is necessary. A direct test for the presence of parasites such as PCR on peripheral blood may be ideal, but for practical purposes, we focused this study on DAT (10). Our recent study in Bangladesh measured an infection-to-disease conversion rate of 5%, and other studies have reported large variance in this rate, ranging from 2% in Nepal to 4% to 38% in Bihar, India (8, 10, 14, 15). While reporting case numbers is pertinent to disease control in affected regions, establishing and monitoring the number of asymptomatic infected individuals is even more critical for control measures to succeed. In this study, we characterize serological tests comprising the rTR18 and rKR95 antigens as suitable for the detection of asymptomatic infected individuals from a region of Bangladesh where VL is hyperendemic. The two antigens are conserved across Leishmania species but, with the exception of T. cruzi, lack homologs in related organisms or organisms prevalent in regions where VL is endemic. ELISAs with the two antigens demonstrated high sensitivity and specificity for asymptomatic infected individuals.

A tool to detect asymptomatic L. donovani infection must be suitable for large population studies in regions where VL is endemic and must be capable of detecting individuals harboring low-grade infection. An easy-to-use and quantitative serological assay detecting L. donovani-specific antibodies will fulfill these criteria. DAT is used extensively in the Indian subcontinent to detect asymptomatic infection. DAT has the advantage of using antigens derived from whole promastigotes, which provides a wide range of antigens for antibodies to bind. But DAT can be hard to standardize for low-grade infections, is prone to reader errors, and is at best semiquantitative. As noted by users, the use of lysed or freeze-dried whole promastigotes as the antigen increases the chances of lot-to-lot variations in performance (22). Given recent observations that DAT titers of ≥6,400 indicate a higher risk of progressing to acute VL disease in the Indian subcontinent, the agreement of the antigens, especially rKR95, with high DAT titers supports their utility in detecting asymptomatic infection (Table 2) (13). The issue of low antibody levels against rTR18, rKR95, and L. donovani SLA in some high DAT-positive samples may be the result of assay performance on fresh versus archived samples. Nevertheless, the agreement seen in this study with DAT is among the highest observed when considering many other studies correlating serological tests with DAT on asymptomatic individuals (10, 14, 16). High levels of antibodies against the antigens in quantitative PCR (qPCR)-positive individuals also indicate their utility in being robust markers of infection.

rK39 is the gold standard antigen for confirming VL disease in the Indian subcontinent, and the rK39 RDT is the clinical confirmatory test recommended by the kala-azar elimination program. It has also been used off-label in regions where VL is endemic for surveillance purposes (8, 23). Given its dedicated role in confirming VL and the knowledge of its capabilities among health workers, use of the rK39 RDT/ELISA to define asymptomatic infection among people with no clinical symptoms of VL may be extremely problematic. A serological test based on a combination with alternative recombinant antigens is hence more suitable as a screening tool. Together the rTR18 and rKR95 antigens can detect most of the rK39 ELISA-positive serum samples as well as some rK39 ELISA-negative serum samples. These observations further indicate their utility in a serological test to identify asymptomatic infected individuals.

Our results suggest that a combination of the rTR18 and rKR95 antigens with rK39 can provide better performance in a standardized test than one developed with either antigen alone. Antigens can be incorporated into quantitative ELISA as well as RDT formats, either of which would be suitable for widespread use in regions where VL is endemic. A combination of antigens can be achieved either through simple mixture or by producing a single recombinant fusion protein incorporating rTR18, rKR95, and rK39. Fusion antigens have been demonstrated to be highly effective in combining the reactivity of their component antigens and can be produced more cost-effectively than the individual recombinant protein components.

An important goal of the ongoing kala-azar elimination program is active detection, which can only be accomplished by periodic surveillance, identification of at-risk individuals, and early detection of VL symptoms followed by treatment. A serological test comprising the rTR18 and rKR95 antigens can be a valuable tool for periodic surveillance and an important first step toward identification of at-risk individuals. While our results are encouraging, a limitation is the small number of samples examined. A larger longitudinal study in regions where VL is endemic is required to further evaluate the utility of rTR18 and rKR95 in detecting asymptomatic infection. Such a comprehensive study would help to accurately report on exposure and progression to disease, numbers that have previously been elusive and difficult to rationalize, but that are absolutely necessary for the success of intervention and elimination programs in regions of VL endemicity.
